# Auditory and Visual Electrophysiology of Deaf Children with Cochlear Implants: Implications for Cross-modal Plasticity

**DOI:** 10.3389/fpsyg.2017.00059

**Published:** 2017-02-01

**Authors:** David P. Corina, Shane Blau, Todd LaMarr, Laurel A. Lawyer, Sharon Coffey-Corina

**Affiliations:** ^1^Cognitive Neurolinguistics Laboratory, Center for Mind and Brain, University of California at Davis, DavisCA, USA; ^2^Department of Linguistics, University of California at Davis, DavisCA, USA

**Keywords:** cross-modal plasticity, cochlear implants, deaf children, intramodal plasticity, developmental p1, developmental n1, ERP

## Abstract

Deaf children who receive a cochlear implant early in life and engage in intensive oral/aural therapy often make great strides in spoken language acquisition. However, despite clinicians’ best efforts, there is a great deal of variability in language outcomes. One concern is that cortical regions which normally support auditory processing may become reorganized for visual function, leaving fewer available resources for auditory language acquisition. The conditions under which these changes occur are not well understood, but we may begin investigating this phenomenon by looking for interactions between auditory and visual evoked cortical potentials in deaf children. If children with abnormal auditory responses show increased sensitivity to visual stimuli, this may indicate the presence of maladaptive cortical plasticity. We recorded evoked potentials, using both auditory and visual paradigms, from 25 typical hearing children and 26 deaf children (ages 2–8 years) with cochlear implants. An auditory oddball paradigm was used (85% /ba/ syllables vs. 15% frequency modulated tone sweeps) to elicit an auditory P1 component. Visual evoked potentials (VEPs) were recorded during presentation of an intermittent peripheral radial checkerboard while children watched a silent cartoon, eliciting a P1–N1 response. We observed reduced auditory P1 amplitudes and a lack of latency shift associated with normative aging in our deaf sample. We also observed shorter latencies in N1 VEPs to visual stimulus offset in deaf participants. While these data demonstrate cortical changes associated with auditory deprivation, we did not find evidence for a relationship between cortical auditory evoked potentials and the VEPs. This is consistent with descriptions of intra-modal plasticity within visual systems of deaf children, but do not provide evidence for cross-modal plasticity. In addition, we note that sign language experience had no effect on deaf children’s early auditory and visual ERP responses.

## Introduction

Congenital deafness leads to significant language delays in children acquiring spoken language. Cascading effects of impoverished linguistic knowledge impact a wide range of psychological and cognitive behaviors including self-regulation (Calderon and Greenberg, 2011), working memory (Pisoni and Geers, 2000), and reading (Perfetti and Sandak, 2000). About two to three out of every 1,000 children in the United States are born with a detectable level of hearing loss in one or both ears (Centers for Disease Control and Prevention, 2010). Cochlear implants (CIs) have become a popular treatment option for deaf children. These devices deliver electrical stimulation to the auditory nerve, bypassing malfunctioning peripheral auditory mechanisms. Deaf children who receive a cochlear implant early in life and engage in intensive oral/aural therapy often make great strides in spoken language acquisition. However, even under optimal conditions and the best efforts of clinicians, there is a great deal of variability in language outcomes (Tobey et al., 2012).

The interplay of factors contributing to this lack of success is poorly understood (Svirsky et al., 2000; Geers et al., 2008; Peterson et al., 2010). One increasing concern is that under conditions of deafness, the auditory system is subject to cross-modal plasticity (CMP), (Kral and Sharma, 2012; Sharma and Mitchell, 2013). In CMP, primary sensory cortices that are associated with a deprived modality can become colonized by the remaining modalities (Bavelier and Neville, 2002). In the case of deafness, the processing demands of an intact sensory system, such as vision, may recruit nascent auditory cortex making it less available for speech processing. The extent to which this has negative effects on auditory processing after implantation, may be referred to as maladaptive CMP.

Early studies of animal models of deafness provide evidence supporting the idea that CMP is present in humans (Allman et al., 2009; Meredith and Allman, 2009). For example Lomber et al. (2010) demonstrate supranormal enhancements in peripheral vision localization and visual motion detection in deaf cats. These enhanced functions are isolated to anatomically distinct auditory regions: primary auditory field (PAF), associated with increased visual peripheral target detection, and the dorsal zone of the auditory cortex (DZ). Critically, the causal relationship between visual and auditory function was demonstrated by selective cooling of auditory association regions resulting in a loss of the supranormal abilities. However, recent evidence suggests that responsiveness to visual input in DZ is in fact quite limited and importantly doesn’t come at a cost of auditory functionality (Land et al., 2016).

Recent work has reported evidence of CMP in pre- and post-lingually deaf adults with CIs which has been suggested to be maladaptive. In studies that have reported maladaptive CMP, the research often makes use of a neural marker of visual processing (e.g., P1 or N1 evoked potentials), and relates this signal to a behavioral processing deficit such as identification of speech in noise (Doucet et al., 2006; Buckley and Tobey, 2011; Sandmann et al., 2012; Campbell and Sharma, 2016; Kim et al., 2016). The inference is then made that the altered visual response is causally related to the auditory speech processing and, by association, that auditory cortical regions are vulnerable to reorganization (Sharma et al., 2015).

There are several weaknesses in this line of reasoning, the foremost of which is that a high-level auditory function like the recognition of words is a multi-component process that encompasses many distinct processing stages. This involves not only fundamental elements of acoustic processing, but mechanisms of speech segmentation, phonemic identification, and lexical recognition, as well as other cognitive properties such as attention to the stimuli, and in the context of multi-modal testing, integration of visual speech information. Thus behavioral performance draws on many cognitive systems that extend beyond the function of primary auditory cortex alone.

A second weakness is that the methods that are used to assert that auditory cortex capabilities have been usurped by visual processing rely on source localization of ERP signals such as sLORETA (Pascual-Marqui, 2002; see for example Sharma et al., 2015). Such methods are known to have limitations with neural data, particularly where there may be simultaneously active sources. A strong or superficial source may obscure weak or deep sources, and nearby sources of similar orientation tend not to be separated but interpreted as one source located roughly in between (Wagner et al., 2004). Caution is further warranted in the context of EEG data collected in the presence of CIs, as it is unclear how device-generated signal and noise may impact spatial resolution and source localization solutions.

Furthermore, few published studies have directly evaluated physiological measures of auditory and visual function in a pediatric population with congenital deafness. The use of pediatric populations is especially important as children in their formative years of language development may be at greatest risk of developing maladaptive CMP. This also highlights the active role of the language acquisition process, as the question has been raised as to whether language input itself may play a role in maladaptive CMP. For instance, Giraud and Lee (2007) assert that “exposure to sign language in the first 3 years of life locks the language system into a vision-only configuration that prevents possible future acquisition of auditory language,” suggesting children exposed to visual language input should be at greater risk for maladaptive CMP.

The present study addresses these concerns by collecting both auditory and visual evoked potentials (VEPs) in a pediatric population in the early and middle stages of language development, including children exposed to sign language and those enrolled in aural/oral-only programs. We begin with an experiment designed to elicit a cortical auditory evoked potential, the auditory P1, which has been described as biomarker of primary auditory cortex development in deafness (Sharma et al., 2015). Next, we turn to a visual experiment designed to elicit VEPs where the onset and offset of a patterned peripheral visual display results in a characteristic biphasic P1–N1 complex. Comparing these measures allows us to examine whether visual processing modulates lower-level auditory function in children with CIs. We further explore whether a subject’s language experience (exposure to oral versus signed language) interacts with the expression of these auditory and visual markers.

## Experiment 1: Auditory Processing

### Materials and Methods

#### Participants

Twenty-six congenitally deaf children with severe-to-profound sensorineural hearing loss, ages 2.0–8.5 years (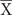 = 4.10), who received CIs, served as subjects. Twenty-five normally hearing children, ages 2.4–8.3 years (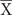 = 5.2), served as controls. **Table 1** presents the subject characteristics and demographics of the deaf children involved in the present study. Shown in **Table 1** is the child’s age at time of testing, age of first implant (in days) (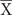 = 701.5, range 287–1581), gender, cochlear implant(s) (bilateral, unilateral), time in sound (in days for the first implant) (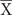 = 1006.65, range 239–2098), and language inventory scores (spoken and sign language production). Auditory data from two subjects (S13 and S22) was corrupted and therefore not used in the analysis of the auditory results.

**Table 1:** Characteristics of deaf subjects in the present study, including age, gender, age at first implantation, whether bilaterally or unilaterally implanted, time since first implantation measured in days (Time in Sound; TIS), and scaled words/signs produced, gathered from a parental report of language production.

Subject	Age	Gender	Age at first Implant	CIs	TIS	Words Prod.	Sign Prod.
1	2.00	M	367	bi	361	44.44	58.89
2	2.01	M	513	bi	264	23.33	76.67
3	2.05	F	646	bi	239	31.11	88.89
4	2.11	M	305	bi	361	15.56	23.33
5	3.02	F	287	bi	893	94.44	0
6	3.02	M	526	bi	713	100	0
7	3.03	M	695	bi	504	23.33	74.44
8	3.06	F	532	bi	749	26.67	85.56
9	3.07	M	536	bi	626	77.78	0
10	3.07	M	290	bi	287	92	0
11	3.10	M	340	bi	1051	77.78	0
12	4.02	F	793	uni	735	89.66	0
13	4.03	F	395	bi	1155	NA	0
14	5.00	F	695	uni	600	92.22	91.11
15	5.01	M	1057	bi	794	88.89	91.11
16	5.02	M	377	bi	1520	92.13	0
17	5.07	M	691	bi	1003	90	0
18	5.08	F	725	bi	1361	93.33	23.33
19	6.03	M	724	bi	1924	100	0
20	6.05	F	585	bi	1800	93.33	0
21	6.09	M	550	bi	1915	88.89	0
22	6.09	M	1581	bi	904	100	51.11
23	7.07	F	1544	bi	1246	90	0
24	7.08	M	1236	bi	1577	84.44	0
25	7.09	M	723	bi	2098	93.33	0
26	8.05	M	1526	uni	1493	100	100

#### Behavioral Testing

Caregivers completed a modified 92 item MacArthur language inventory for English (Fenson et al., 1994) and American Sign Language (Anderson and Reilly, 2002).

#### ERP Testing

Children were fitted with a 22-channel electrode cap. Most children sat in an appropriately sized chair, while some younger participants sat on their parent’s lap during recording. In all cases, an experimenter sat to the right of the child. During the auditory testing, children were seated in front of Dell Latitude 620 laptop computer and watched a silent cartoon or played an iPad game (sound muted) while auditory stimuli were presented.

Auditory stimuli were presented in an oddball paradigm designed to elicit a P1 cortical auditory evoked potential. The stimuli consisted of either a synthesized speech syllable (/ba/) which served as a standard (85%) or a frequency modulated tone (600–1200 Hz) which served as a deviant (15%), both of which were 100 ms in duration. Auditory stimuli were presented free-field at 65 db for deaf children and 60 db for hearing controls using AUVIO 05A13 speakers located approximately 45° degrees to left and right of the subject and powered by a NuForce Icon amplifier driven by the laptop’s audio output. Stimuli presentation was jittered between 2 and 4 s to reduce expectancies. A total of 202 trials per subject were presented, lasting approximately 4.5 min.

#### Data Recording and Analysis

ERPs were collected using a Biosemi Active Two recording system (Biosemi B. V., Amsterdam, Netherlands). Recordings were taken at 22 electrode sites, using standard 10/20 system. Three additional external electrodes were used to record data from left and right mastoids and the third was placed below the left eye to monitor eye movements. The eye electrode was used to assist in eliminating trials where blinks or horizontal eye movements occurred and trials where participants were looking away. Voltage offsets between each active electrode and CMS (common mode sense -the online reference) were below 20 μV, before the start of data collection. Offsets were checked again at the end of the recording session.

Sampling rate during recording was 512 Hz. Oﬄine, continuous data was downsampled to 256 Hz, and bandpass filtered at 0.1–30 Hz. Data from scalp and eye electrodes were re-referenced oﬄine to the average of left and right mastoids. Initial analysis of the EEG data was performed using the ERPLAB plugin (Lopez-Calderon and Luck, 2014) for EEGLAB (Delorme and Makeig, 2004). Independent Component Analysis (ICA) using the Infomax algorithm implemented in the EEGLAB Toolbox was used to remove both eye movement and cochlear implant artifacts. ICA analysis was performed on both auditory and visual data in order to reduce eye blink artifact. Between one and two components were removed due to eye blink. Four subjects (2–3 year olds) did not have any obvious eye blink components in their visual data. In these cases, no components were removed. In all subjects, additional artifact rejection was performed automatically, removing all trials where voltage exceeded ±100 μV, in all channels that were used in analysis. For the auditory data 12.6% (range 0–47.6%, *SD* 10.85) of deaf subjects trials were rejected, while 15.07% (range 0.7–32.9%, *SD* 10.1) of hearing children’s trials were rejected. *T*-test indicated that the numbers of auditory trails rejected across groups did not differ (*t* = 0.83, *p* = 0.42). For visual data 10.72% (range 0–42.6%, *SD* 10.09) of deaf subjects trials were rejected, while 8.6% (range 0–28.8%, *SD* 8.4) of hearing children’s trials were rejected. *T*-test indicated that the number of visual trails rejected across groups did not differ (*t* = 1.13, *p* = 0.26).

Auditory data was collected with CIs functioning. In 11 children with CI, we were unable to establish contact at lateral temporal or parietal sites due to the location of the implanted receiver/stimulator. In these cases, we eliminated the affected channels prior to data analysis. ICA analysis was used to remove CI artifact from the deaf participant data. Between 1 and 5 components per subject were removed in auditory data set. Auditory data reported here represent responses only to the standard /ba/ stimuli. For all analysis, automatic peak detection [most positive (P1) or negative peak (N1)] was taken using ERPLAB’s, ERP measurement tool (Lopez-Calderon and Luck, 2014).

#### Statistical Analysis

Statistical analyses of peak amplitude and latency values used mixed effects models which were estimated using the lme4 package (Bates et al., 2015) in R (R Core Team, 2016). Mixed effect models offer many advantages over traditional ANOVAs, including simpler *post hoc* testing, better modeling where assumptions of sphericity are violated (i.e., unequal variances across subjects), and analyses that are robust in cases with missing data and where cells are not completely balanced (Gueorguieva and Krystal, 2004). Increasingly mixed effect models are being used to evaluate EEG activity (see, for example Payne et al., 2015).

The group-level models included factors of Group, Age, and Gender.

All models were initially estimated with the maximum available fixed effects structure with factors iteratively assessed for significance. Individual factors were removed by excluding the factor with the lowest *t*-value and refitting the model until only factors with a *t*-value above 2 remained. Each model was also fitted with by-subject and by-site (frontal midlines sites Fz and Cz) random intercepts.

### Results

**FIGURE 1:**
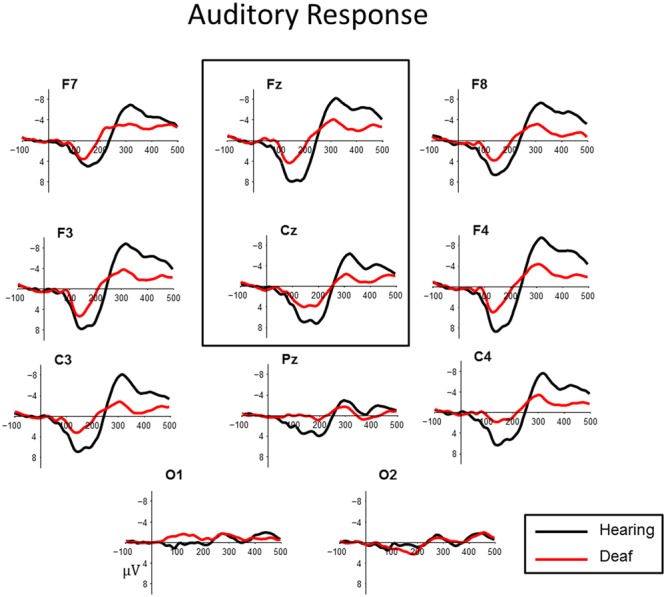
**Auditory cortical evoked potentials for deaf (red) and hearing control (black) groups at central midline sites (Fz, Cz).** For illustrative purposes, representative data from sites (Pz), left and right frontal (F7, F8, F3, F4) and lateral temporal sites (C3, C4) and occipital sites (O1, O2) have been included.

In control subjects, the electrophysiological response to auditory standards produced a positive peak between 100 and 200 ms, followed by a negative peak around 300 ms post stimulus. This was most prominent over fronto-central sites (**Figure 1**). This morphology is consistent with cortical auditory evoked potential P1–N1 complex. Deaf children showed more variable responses both in latency and in waveform morphology. To quantify the observed patterns across groups we measured both peak amplitude and latency of the most positive peak between 70 and 175 ms at frontal midlines sites Fz and Cz for all subjects.

#### Auditory P1 Amplitude

A main effect of Group indicated that hearing controls showed a larger P1 compared to the deaf children with CIs (*t* = -2.424, *p* = 0.019; Hearing 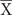 = 8.36 μV, Deaf 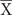 = 5.73 μV). This effect is illustrated in **Figure 1**. No other factors were significant.

#### Auditory P1 Latency

**FIGURE 2:**
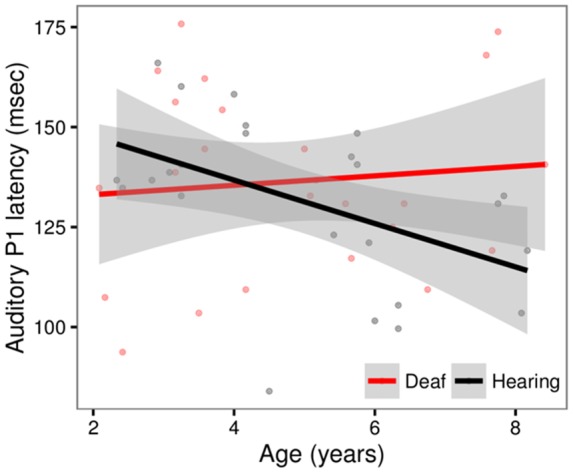
**Scatterplot showing the relationship between Age and P1 latency for deaf children with cochlear implants (red) and hearing controls (black), in sites Fz and Cz.** The solid line shows the linear regression for each group, with standard error represented by the gray band. In the hearing control group, P1 latencies decrease with age. This pattern is not observed in the deaf group.

We observed a main effect of Age (*t* = -2.29, *p* = 0.03) and a significant Age × Group Interaction (*t* = 2.00, *p* = 0.05). The Age × Group interaction is depicted in **Figure 2**. The scatter plot shows that while hearing children show expected age-related changes (latencies decrease with age), this pattern is not observed in the deaf children with CIs (Hearing, *r* = -0.46, *t* = -3.53, *p* = 0.001; Deaf, *r* = 0.09, *t* = 0.59, *p* = 0.56). No other factors were significant.

### Discussion

The auditory experiment was successful at eliciting an identifiable P1 auditory evoked potential in the majority of control children and deaf subjects with CIs. The P1 auditory evoked potential reflects the sum of the accumulated synaptic delays and neural conduction times as an auditory signal travels from the ear to the primary auditory cortex. Gilley et al. (2008) report that the cortical generator of the P1 is the auditory cortex in normal hearing children. However, the amplitude of the P1 has been shown to be sensitive to stimulus level (Bertoli et al., 2011), thus the amplitude difference between hearing and deaf children observed here may reflect the reduced perceived signal intensity in the children with CIs.

P1 latency has been used as a biomarker of auditory system maturity. The latency of the P1 has been shown to decrease with age in normal hearing children (Eggermont, 1988; Liegeois-Chauvel et al., 1994; Eggermont et al., 1997; Sharma et al., 1997, 2002). In our data, hearing control children show these expected age-related changes while this pattern is not observed in deaf children. In previous work with deaf children implanted with CIs prior to 3.5 years old, Sharma et al. (2002) showed normal P1 latency and morphology by 7–8 months post implant. Our data in part support this observation, however, we do note that four of the children who received a cochlear implant prior to 3.5 years and have had at least 8 months experience with their CI show a longer than expected P1 latency based upon the published norms (Sharma et al., 2002). These data suggest that even with early implantation and adequate experience, some children with CIs will nevertheless exhibit atypical P1 latencies, potentially reflecting an aberrant maturation of cortical function.

## Experiment 2: Visual Processing

To investigate visual function in our cohort, subjects were asked to watch a silent cartoon presented in the center of a laptop screen, while a checkerboard pattern was intermittently displayed in the peripheral surround. The appearance of the checkerboard results in a robust visual “onset” evoked potential. Similarly, the disappearance of this patterned display often yields a secondary visual “offset” evoked potential. Using this paradigm we investigated the visual responsivity of hearing controls and deaf children with CIs. To the extent that CMP is evident in our deaf sample, we might expect to see VEPs that are qualitatively different from hearing controls.

### Materials and Methods

#### Participants

The same deaf and hearing subjects participated in the visual experiments as in Experiment 1. Deaf children had their CIs turned off during the visual experiment. Both auditory and visual testing was done for all subjects at the same time (one ERP testing session for each subject).

#### Procedures

Following participation in the passive auditory task, subjects watched a silent cartoon presented in the middle of the screen against a dark gray background. A radial black and white checkerboard (24 checks/6 annular rings, subtending 21.24° visual angle) intermittently replaced the background and lasted for 2, 3, or 4 s. There were a total of 60 trials, which lasted approximately 6 min. This peripheral visual stimulus was designed to elicit a pattern-onset and a pattern-offset VEP. Based on previous reports of differences between deaf and hearing subjects observed during visual processing we focused on the expression of the P1 and N1 visual components (Buckley and Tobey, 2011; Sandmann et al., 2012; Campbell and Sharma, 2016).

#### Data Recording and Analysis

EEG procedures and analysis are identical to that of Experiment 1.

#### Statistical Analysis

Statistical analyses of peak amplitude and latency measures followed the same procedures used in Experiment 1. Here, the Group-level model included fixed effects of Pattern (onset/offset), Group, Age, and Gender, as well as by-subject and by-site (O1, O2, Pz, and fronto-central sites Cz and Fz.) random effects. *Post hoc* testing used Tukey’s HSD, corrected for multiple comparisons, implemented by the lsmeans package (Lenth, 2016) in R.

### Results

**FIGURE 3:**
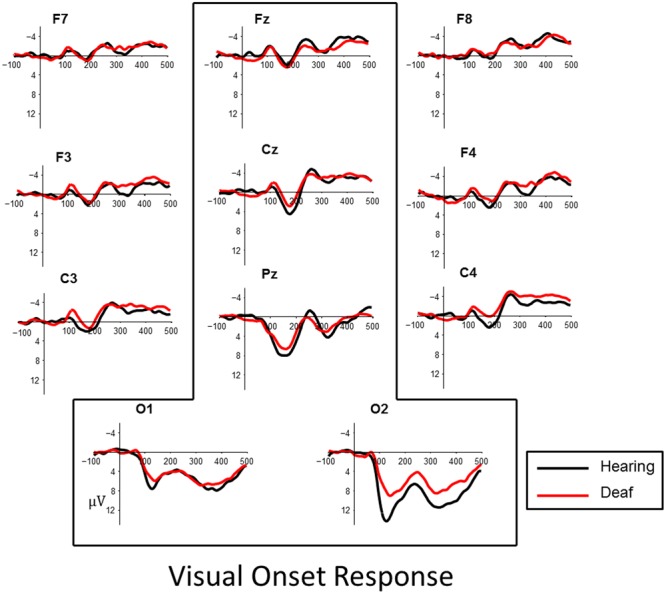
**Visual evoked responses for pattern-onsets at central midline sites (Fz, Cz, Pz) and occipital sites (O1, O2) for deaf (red) and hearing (black) groups.** For illustrative purposes, representative data from sites left and right frontal (F7, F8, F3, F4) and lateral temporal sites (C3, C4) have been included.

**FIGURE 4:**
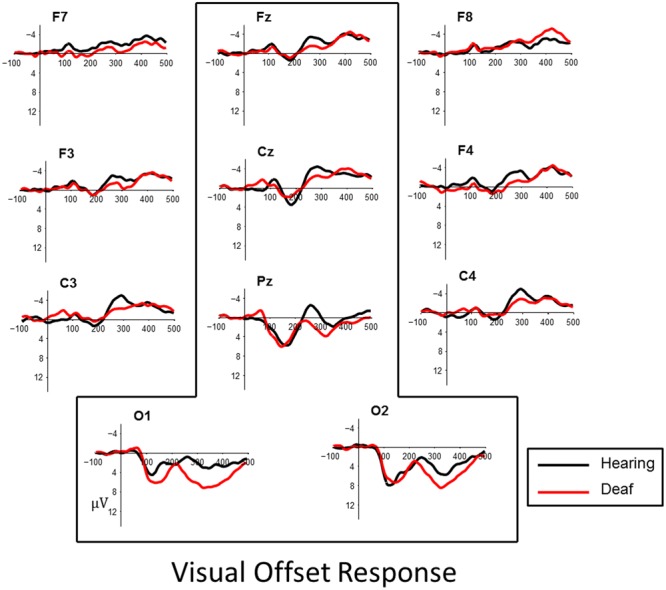
**Visual evoked responses for pattern-offsets at central midline sites (Fz, Cz, Pz) and occipital sites (O1, O2) for deaf (red) and hearing (black) groups.** For illustrative purposes, representative data from sites left and right frontal (F7, F8, F3, F4) and lateral temporal sites (C3, C4) have been included.

Visual inspection of the data revealed a P1, peaking at approximately 165 ms followed by an N1, peaking about 250 ms, in both onset and offset of the checkerboard pattern. The effects were most robust at posterior parietal and occipital sites (**Figures 3** and **4**). To quantify observed VEP differences we examined the window of 70–200 ms post stimulus to characterize effects related to the P1 component. The window of measurement used for the N1 was 175–325 ms. Analysis of the visual data included posterior electrode sites O1, O2, Pz, and fronto-central sites Cz and Fz.

Automatic peak detection [most positive (P1) or negative peak (N1)] was taken using ERPLAB’s measurement tool (Lopez-Calderon and Luck, 2014).

#### Visual P1 Amplitude

Examining data from the visual VEP responses we find a main effect of Pattern, showing overall larger responses to pattern onsets than offsets (*t* = -2.58, *p* = 0.01; Hearing: 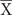 onsets = 8.83 μV, 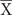 offsets = 6.55 μV; Deaf: 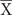 onsets = 7.88 μV, 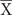 offsets = 7.26 μV).

No other factors were significant predictors of P1 amplitude, and *post hoc* testing showed no significant difference between Group for onset amplitude (*t* = 0.97, *p* = 0.33) or offset amplitude (*t* = -0.66, *p* = 0.51).

#### Visual P1 Latency

**FIGURE 5:**
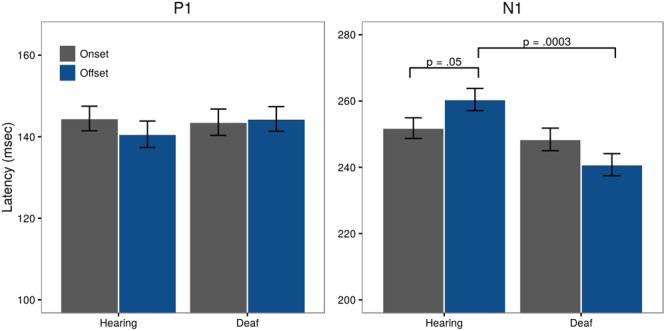
**Latencies for visual P1 and N1 for both onset (gray) and offset (blue) responses, collapsed across the sites included in the analysis (Fz, Cz, O1, Pz, O2)**.

Looking at P1 latencies, we find no significant differences between onset and offset within Groups (*t* = -1.18, *p* = 0.24; Hearing: 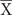 onsets = 144.47 ms, 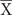 offset = 140.59 ms; Deaf: 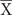 onsets = 143.55 ms, 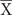 offsets = 144.35 ms). *Post hoc* testing revealed no significant differences between Group for pattern offset (*t* = -0.73, *p* = 0.47) or onset latencies (*t* = 0.18, *p* = 0.86) (**Figure 5**, left panel).

#### Visual N1 Amplitude

Examining visual N1 amplitude, we observed a trend in Pattern which showed that N1 was larger to offset relative to onset in both groups (*t* = -1.88, *p* = 0.06; Hearing: 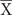 onsets = -2.70 μV, 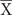 offset = -4.09 μV; Deaf: 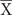 onsets = -2.06 μV, 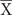 offsets = -2.47 μV). *Post hoc* testing showed no significant amplitude difference between Groups for either onsets (*t* = -0.57, *p* = 0.57) or offsets (*t* = -1.50, *p* = 0.14).

#### Visual N1 Latency

Assessment of N1 latency showed a significant difference in Pattern for the control group with offset latencies longer than onset latencies (*t* = -1.95, *p* = 0.05; 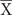 onsets = 251.82 ms, 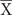 offset = 260.45 ms). A significant Pattern × Group interaction (*t* = -2.64, *p* = 0.008) showed that in deaf subjects, offset latencies were shorter than onset latencies (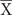 onsets = 248.41 ms, 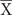 offsets = 240.78 ms). *Post hoc* testing further revealed a significant Group difference for offset latencies (*t* = 3.73, *p* = 0.0003), but not onsets (*t* = 0.66, *p* = 0.51). These differences are illustrated in **Figure 5**, right panel.

### Discussion

The visual data reveal no robust group differences in P1 VEP amplitude or latency. Previous studies examining the P1 VEP in adult deaf and hearing participants have reported mixed results. In early work conducted by Neville and Lawson (1987a), there were no reported differences in P1 VEP latency or amplitude in deaf adult subjects compared to hearing controls in a peripheral motion detection task. Armstrong et al. (2002) reported no group differences between deaf and hearing adult subjects in P1 amplitude or latency in response to sinusoidal gratings presented in the fovea and peripheral visual field. Doucet et al. (2006) recorded VEP to shape-changing stimuli in deaf adults with CIs and hearing controls. No group differences were found for either P1 latency or amplitude. Using the same shape-changing stimuli used by Doucet et al. (2006) and Campbell and Sharma (2016) reported no differences in early P1 latency and amplitude in a comparison of deaf children with CIs and hearing controls.

In contrast, Sandmann et al. (2012) reported VEPs to parametrically varied flashing checkerboard stimuli in a heterogeneous group of post-lingual deafened adults (mean age 54, range 38–70 years) who received CIs as adults. They reported reduced P1 amplitudes and shorter latencies in the CI group relative to hearing controls, which they interpreted as indexing a different degree of visual cortex recruitment in CI users compared to controls. They speculated this reduced latency may reflect shorter, more efficient visual information processing. Hauthal et al. (2014) examined VEPs in congenitally deaf and hearing subjects to reversing checker-board stimuli that were systematically modulated in luminance ratio. These participants showed similar modulation of VEP amplitudes (N85, P110) and latencies (P110) to the luminance modulation. However, compared to hearing subjects, deaf participants showed shorter N85 latencies and larger P110 amplitude. These findings are taken to suggest an indication of more efficient neural processing of visual information in the deaf.

Bottari et al. (2011) reported that in response to a visual warning signal, deaf subjects showed a decreased latency in the C1 (45–95 ms) and differential P1 morphology compared to controls. On the other hand, responses to visual targets resulted in longer P1 latencies in deaf compared to hearing controls and P1 amplitude in deaf subjects was correlated with reaction time performance on their task. Bottari et al. (2011) suggest changes in the P1 dynamics in the deaf may thus reflect stronger exogenous attention capture in deaf compared to hearing subjects.

In contrast to the P1, the N1 data show a stronger group difference for offset VEP responses. Specifically we see a shorter N1 latency in the deaf subjects compared to the hearing subjects. Previous research on later visual components, including the N1, show that these components are more consistently modulated by deafness than the visual P1.

Neville and Lawson (1987a) reported larger attention-related N1 modulations over occipital regions and left-temporal and parietal regions in deaf subjects compared to hearing controls. Armstrong et al. (2002) reported deaf participants showed larger N1 amplitudes to central and peripheral movement stimuli. Buckley and Tobey (2011) examined the N1 VEP response to peripheral movement targets in two groups of deaf subjects with CIs. These subjects either had pre- or post-lingual onset of severe-to-profound hearing loss. They found that larger N1 amplitude was associated with lower speech perception scores in prelingually deaf subjects. This pattern was not observed in subjects with post-lingual deafness. Campbell and Sharma (2016) reported some evidence of larger N1 amplitude and earlier latencies in deaf children with CIs, a pattern similar to our own data which we observe in offsets only. It is interesting to note that in the present experiment, group differences were observed in response to a static visual image display, rather than the more commonly used dynamic movement stimuli.

It is well known that attention may modulate N1 VEP (Clark et al., 1995; Mangun, 1995). We find it noteworthy that N1 latency effects were observed to the offset of the visual stimulus. This may indicate that deaf children with CIs are more attentionally vigilant to visual stimulus, where attentional capture may be triggered for both the appearance and disappearance of a visual stimulus. Further enhancements may be evident for dynamic movement stimuli.

## Relationship Between Auditory and Visual Data

To date studies of cross-modal interactions in deaf individuals with CIs have typically reported broad correlations between VEPs and behavioral measures of speech understanding (e.g., Doucet et al., 2006; Buckley and Tobey, 2011; Sandmann et al., 2012; Kim et al., 2016). In the present study, we have the ability to examine more directly the relationship between auditory and visual activity. Specifically, we questioned whether there was a relationship between auditory P1 latency and visual N1 offset latency within subjects. Recall, in our data the deaf subjects showed the expected lack of developmental progression in their auditory P1 latencies, suggesting an aberrant maturation of cortical function. Here, we ask whether the degree of variance associated with auditory P1 latencies in our CI subjects is accounted for by visual reactivity as indexed by our visual-offset N1 measure.

### Materials and Methods

#### Participants

The data obtained from deaf and hearing subjects who participated in Experiments 1 and 2 were included in this analysis.

#### Procedures

We examine visual data from two electrode sites, site O2 where we observed our largest N1 latency difference, and further examine the visual response at Cz. Central site Cz was chosen as this site is typically associated with a robust auditory P1–N1 response and is thought to reflect synchronous neural activation of structures in the thalamic-cortical segment of the central nervous system in response to auditory stimulation (Vaughan and Ritter, 1970; Wolpaw and Penry, 1975; Naatanen and Picton, 1987; Woods, 1995). Some caution is warranted in the comparison of the N1 response recorded from site O2 and the N1 response recorded at site CZ, as these may reflect difference sources (Coch et al., 2005).

#### Statistical Analysis

In an effort to establish a relationship between auditory and visual evoked responses, we constructed two models of auditory P1 latency that included factors of Group, Age, Gender, and N1 latency. This latter measure contained each subject’s N1 latency value at electrode site Cz in the first model, and in the second model, at electrode site O2 where the largest response was observed. As before, both models also included random intercepts for each subject and electrode site.

### Results

**FIGURE 6:**
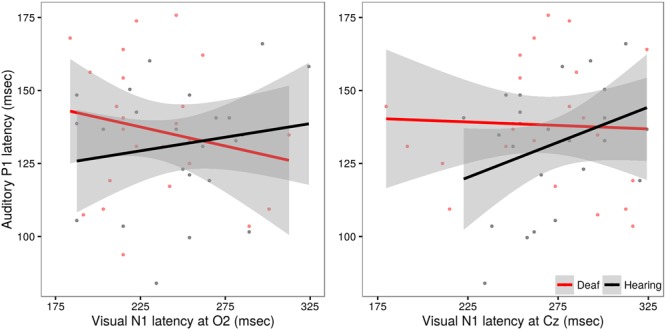
**Illustration of the relationship between auditory P1 latency and visual N1 data in deaf (red) and hearing (black) groups.** Auditory P1 latencies are collapsed across channels Cz and Fz. In the left panel, VEP data from site O2, in the right panel, VEP data from site Cz. The solid line shows the linear regression for each group, with standard error represented by the gray band. Hearing children exhibit a significant positive relationship between auditory and visual latencies responses recorded at site Cz.

In evaluating these models, no significant effects beyond those already discussed were observed (all *p*-values >0.20). A *post hoc* analysis using Pearson’s product-moment correlation provides further confirmation of the lack of relationship between auditory and visual latencies observed in deaf subjects at site O2 (*r* = -0.17, *t* = -1.19, *p* = 0.24) and at site Cz (*r* = -0.04, *t* = -0.27, *p* = 0.79). However, in hearing subjects, a significant correlation between auditory and visual latencies is observed at Cz (*r* = 0.31, *t* = 2.20, *p* = 0.03), but not O2 (*r* = 0.15, *t* = 1.06, *p* = 0.29). These differences are illustrated in Figure 6.

### Discussion

The finding of a significant correlation between auditory evoked P1 latency and visually evoked N1 recorded at Cz in the hearing children was unexpected. Both early auditory and visual evoked potentials are known to undergo developmental changes, including reductions in latencies from birth through early childhood (Barnet et al., 1980; Lippe et al., 2009). If maturation alone were driving these correlative effects, we would reason that this relationship should be most robust when signals are recorded from sites that maximally capture the sensory effect of interest (e.g., auditory; Cz, visual; O2). However, in the present study we captured a correlation between visual and auditory signals recorded from central site Cz. It is possible that this reflects a more robust coupling between sensory areas in typically developing hearing children, one that is not observed in children with CIs. As noted, the N1 signals recorded at O2 and Cz may reflect different generators (Coch et al., 2005) and the apparent within subject latencies differences across O2 and Cz in our data reinforce this possibility. Additional work is needed to fully characterize these patterns.

## Effects of Language Exposure

The role signed language exposure may play in maladaptive CMP is both controversial and understudied. We wished to explore the relationship between sign exposure, and other demographic variables, and response latency differences observed in the models above.

### Participants and Materials

The data obtained from deaf subjects who participated in Experiments 1 and 2 were included in this analysis. We included subject Age, Gender, Signs and Words Produced (derived from our modified MacArthur inventories), Age of Implantation (of the first implant, in cases of bilateral implantation), and Time in Sound (measured by days since implantation).

### Statistical Analysis

We constructed two new models using only the deaf CI data, and modeled auditory P1 (sites Fz, Cz) and visual N1 response latencies (sites Fz. Cz, Pz, O1, and O2) with factors for Age, Gender, Signs and Words Produced (derived from our modified MacArthur inventories), Age of Implantation (of the first implant, in cases of bilateral implantation), and Time in Sound (measured by days since implantation). Measures of language production, as well as age of implantation, and time in sound, were highly correlated with chronological age. To avoid colinearities in the data, these were each residualized against Age. Both models also included random effects for subject and site, as in previous analyses.

### Results

We found no differences in response latencies as a function of language exposure, signs produced, words produced, age of implantation, or time in sound, in either of the latency models (all *p*-values were >0.25).

### Discussion

While some researchers have questioned whether a deaf child’s mode of language exposure may differentially affect visual and auditory neural systems, with visual sign language experience fundamentally altering auditory language system (Giraud and Lee, 2007), we observed no differences in the auditory and visual responses of deaf children who have been exposed to sign language and those who have elected an oral-based rehabilitative strategy.

## General Discussion

Several findings emerge from these studies. Replicating previous reports, measures of the auditory P1 in deaf children with CIs show morphological patterns that differ from hearing controls. In the present data, the deaf children’s auditory P1 amplitudes were reduced, which may reflect differences in perceived intensity of the stimuli. Auditory P1 latencies were also reduced, and did not show evidence of expected maturational changes observed in hearing controls. Even though the majority of our subjects received a CI before the age of 3.5 years, we observed that some children with CIs will nevertheless exhibit atypical P1 latencies, potentially reflecting atypical maturation of cortical function.

Data from the visual experiment revealed robust latency differences in the N1 components. Deaf children with CIs showed shorter N1 latencies compared to hearing controls in response to the offset of a patterned checkerboard. This distinction in visual responsivity may reflect the plasticity of the visual system of deaf children who have experienced a delay in auditory habilitation.

We evaluated the presence of cross-modal reorganization by examining the relationship between the auditory P1 and visual N1 responses in deaf subjects and hearing subjects. Research has suggested that under conditions of auditory deprivation, regions of auditory cortex may become responsive to visual information at the expense of auditory processing. Evaluating the relationships of the auditory P1 to visual evoked activity in occipital site (O2) and central site (Cz) showed no systematic relationship between evoked-potential latencies across these two sensory domains in the deaf subjects. We observed that variability associated with auditory P1 latencies was not effectively modulated by a high-contrast visual pattern in deaf children. Our data would indicate that auditory cortex does not become responsive to the low level visual signals induced by the stimuli used here as a result of early auditory deprivation in children who have received a cochlear implant early in life. The lack of a trade-off between auditory and visual processing at this level accords with physiological data from deaf cats that show functional changes in visual processing does not come at the cost of auditory function (Land et al., 2016). These data help to delimit the neurophysiological interactions that may be evidenced in the face of auditory deprivation.

It is interesting to note that we did observe a relationship between auditory P1 latencies and visual N1 latencies recorded at central site Cz but not at occipital site O2 for the hearing children. These data may reflect a coupling between auditory and visual sensory systems that is present in typically developing hearing children that is not observed in the deaf children. Further work is needed to understand the development and scope of such interactions.

Finally, we assessed whether the observed latency differences in the auditory P1 and visual N1 components were affected by early exposure to a signed language, age at first implantation, or time since implantation. None of these factors were shown to influence auditory P1 or visual N1 latencies. We especially note that we observed no differences in the auditory and visual responses of deaf children who have been exposed to sign language and those who have elected an oral-based rehabilitative strategy. These data directly challenge claims that exposure to a visual language in the formative stages of language acquisition in deaf children with CIs locks the language system into a vision-only configuration that prevents possible future acquisition of auditory language (Giraud and Lee, 2007).

## Ethics Statement

Statements involving human subjects. This study was carried out in accordance with the recommendations of UC Davis Human Subject Institutional Review Board following the Human Research Protection Program Plan. The protocol was approved by the Human Subject Institutional Review Board. Written informed consent was acquired from the parents of all subjects prior to beginning the experiment. This study involved hearing and deaf children 2–8 years old. Parents were informed that they could stop testing at any time and their children were not required to participate. All subjects gave written informed consent in accordance with the Declaration of Helsinki.

## Author Contributions

DC and SC-C designed the experiment. TL and SB created in-house behavioral measures. DC, SC-C, TL, and SB collected the data. LL did the statistical analysis of the data. SC-C analyzed the ERP data. DC, SC-C, and LL drafted the manuscript.

## Conflict of Interest Statement

The authors declare that the research was conducted in the absence of any commercial or financial relationships that could be construed as a potential conflict of interest.
